# Multivariate Analyses of Small Theropod Dinosaur Teeth and Implications for Paleoecological Turnover through Time

**DOI:** 10.1371/journal.pone.0054329

**Published:** 2013-01-23

**Authors:** Derek W. Larson, Philip J. Currie

**Affiliations:** 1 Department of Biological Sciences, University of Alberta, Edmonton, Alberta, Canada; 2 Department of Biological Sciences, University of Alberta, Edmonton, Alberta, Canada; Monash University, Australia

## Abstract

Isolated small theropod teeth are abundant in vertebrate microfossil assemblages, and are frequently used in studies of species diversity in ancient ecosystems. However, determining the taxonomic affinities of these teeth is problematic due to an absence of associated diagnostic skeletal material. Species such as *Dromaeosaurus albertensis*, *Richardoestesia gilmorei,* and *Saurornitholestes langstoni* are known from skeletal remains that have been recovered exclusively from the Dinosaur Park Formation (Campanian). It is therefore likely that teeth from different formations widely disparate in age or geographic position are not referable to these species. Tooth taxa without any associated skeletal material, such as *Paronychodon lacustris* and *Richardoestesia isosceles*, have also been identified from multiple localities of disparate ages throughout the Late Cretaceous. To address this problem, a dataset of measurements of 1183 small theropod teeth (the most specimen-rich theropod tooth dataset ever constructed) from North America ranging in age from Santonian through Maastrichtian were analyzed using multivariate statistical methods: canonical variate analysis, pairwise discriminant function analysis, and multivariate analysis of variance. The results indicate that teeth referred to the same taxon from different formations are often quantitatively distinct. In contrast, isolated teeth found in time equivalent formations are not quantitatively distinguishable from each other. These results support the hypothesis that small theropod taxa, like other dinosaurs in the Late Cretaceous, tend to be exclusive to discrete host formations. The methods outlined have great potential for future studies of isolated teeth worldwide, and may be the most useful non-destructive technique known of extracting the most data possible from isolated and fragmentary specimens. The ability to accurately assess species diversity and turnover through time based on isolated teeth will help illuminate patterns of evolution and extinction in these groups and potentially others in greater detail than has previously been thought possible without more complete skeletal material.

## Introduction

Vertebrate turnover and diversity approaching the end-Cretaceous mass extinction has been the subject of many recent studies [1]–[7]. Taxa such as large-bodied dinosaurs [8], [9], turtles [10], [11], and amphibians, fish, mammals, and reptiles known from vertebrate microfossil localities [12], [13] have good fossil records in the Upper Cretaceous leading up to the terminal Cretaceous mass extinction. However, turnover and diversity patterns in small-bodied dinosaurs, and particularly small theropod dinosaurs, are not well-understood despite being well-represented in vertebrate microfossil localities by dental remains.

Small theropods have often been identified based on isolated, shed tooth crowns. In North America, Currie et al. [14] examined theropod teeth from the late Campanian Dinosaur Park Formation and used associated skeletal remains to confidently identify specimens. Originally, the study was intended to include teeth from the geographically similar but younger latest Campanian Horseshoe Canyon and Maastrichtian Scollard formations. However, during the course of the study, the authors realized that there were subtle differences between morphologically similar teeth from different stratigraphic levels. Consequently, they restricted the study to Dinosaur Park Formation teeth that had associated skeletal material. They also cautioned that there was probably considerable convergent evolution of tooth form in theropods from different geographic or temporal positions. Nevertheless, based on Currie et al. [14], subsequent workers (eg. [8], [15]–[19]) have identified shed theropod teeth from other formations to the level of species without reference to associated skeletal material from the same host formations, usually because such comparative material does not exist. As well, this problem of identification of theropod taxa from isolated teeth is not restricted to North America. Numerous studies from Africa, Asia, Europe, and South America [eg. 20–40] illustrate that the problem is worldwide. Studies based on more abundant, well-preserved skeletons of other dinosaurs, such as ceratopsians, hadrosaurids, and tyrannosaurids, hypothesize rapid (on the order of 500 thousand to 4.6 million years) dinosaur faunal turnover between and even within formations during the Late Cretaceous [41]–[44]. Based on these hypotheses, it seems unlikely that a single species of small theropod existed for several million years in temporally and geographically disparate formations.

Previously, canonical variate analysis (CVA) has been used with moderate success in quantitatively identifying shed theropod teeth [27], [45], [46], but is much more successful with fewer categories and smaller sample sizes. A similar method, discriminant function analysis (DFA), is used when only two groups are compared [47], and is used in this study to clarify the differentiation of many different taxa with relatively few variables.

In the current study, small theropod teeth from ten lithostratigraphic units in western North America (representing the last 18 million years of the Mesozoic [83.5 to 65.5 Ma]) were compared using CVA and pairwise DFA. The purpose was to test whether or not isolated teeth are quantitatively diagnostic and referable to the few named species known from more complete skeletal material, namely *Dromaeosaurus albertensis*, *Richardoestesia gilmorei*, *Saurornitholestes langstoni*, and *Troodon formosus*. The fossil deposits of western North America provide one of the most continuously preserved and thoroughly sampled terrestrial ecosystems of this time anywhere in the world [48], and as such, provide the best testing ground for comparisons of this nature. The goal was to assess taxonomic diversity through time and determine whether quantifiable assemblage turnover can be documented in the fossil record for this poorly represented group. Such identifications have implications for reconstructing paleocommunities in the absence of better-preserved specimens and establishing the patterns of assemblage composition through the Late Cretaceous fossil record leading up to the end-Cretaceous mass-extinction.

## Materials and Methods

### Measurements

**Figure 1:**
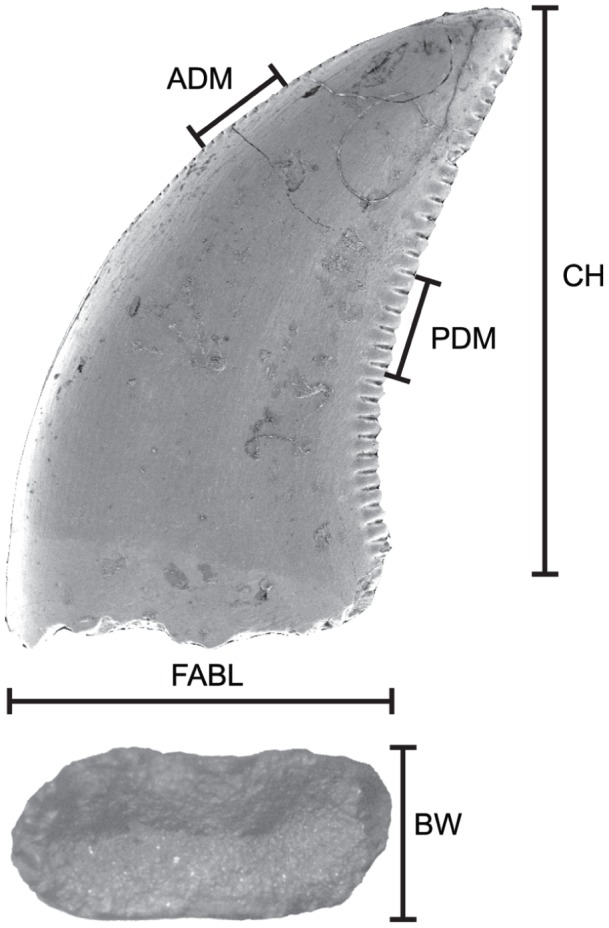
Tooth measurements used in this study. ADM, anterior denticles per millimetre; BW, basal width; CH, crown height; FABL, fore-aft basal length; and PDM, posterior denticles per millimetre.

Measurements were collected from 1183 complete teeth from ten Upper Cretaceous formations (Table S1) representing four time-slices equivalent to the Aquilan (<83.5–80.0 Ma, latest Santonian to early Campanian), Judithian (80.0–75.0 Ma, middle to late Campanian), Edmontonian (72.8–66.8 Ma, late Campanian to Maastrichtian), and Lancian (66.8–65.5 Ma, latest Maastrichtian) North American Land Mammal Ages [44], [49]. Previously published measurement data were taken from Park [29], Sankey et al. [50], [51], Currie and Varricchio [52], Smith et al. [45], Larson [46], and Sankey [53]. Unpublished raw measurements obtained from the authors of Farlow et al. [54] and Longrich [55] were also used, as well as original measurements by one author (DWL) and some previously collected but unpublished measurements by the other author (PJC; Table S1). Principal measurements included fore-aft basal length (FABL), crown height (CH), basal width (BW), and posterior denticles per millimetre (PDM) or their closest approximation if different measurements were employed in the literature (Fig. 1). For example, CH has also been measured from the gumline [50], although this can result in inconsistencies (see Buckley et al. [56] for reasoning). Anterior denticles per millimetre density (ADM) is rarely reported and was used only when possible and if necessary to provide a comparable sample. ADM and PDM, in this study, were analyzed as counts per millimetre (eg. [14], [46], [50]), although this density is sometimes expressed per five millimetres [45] or per ten millimetres [22]. In some instances, a subset of the variables (CH, FABL, and PDM) was analyzed due to lack of published measurements. Tooth measurements were log-transformed to better reflect a normally distributed multivariate dataset.

### Institutional Abbreviations

AMNH–American Museum of Natural History; CMNFV–Canadian Museum of Nature; LSUMGS–Louisiana State University Museum; TMP–Royal Tyrrell Museum of Palaeontology; UALVP–University of Alberta Laboratory for Vertebrate Paleontology; UCMP – University of California Museum of Paleontology; UMNH–Utah Museum of Natural History.

### Analytical Methods

**Figure 2:**
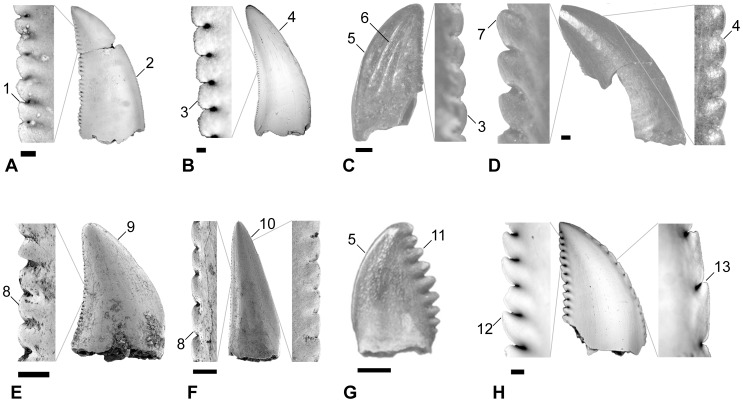
Qualitative morphotypes used to construct a priori categories within formations and the qualitative characters that define them. A, Saurornitholestinae; B, Dromaeosaurinae; C, cf. *Zapsalis*; D, Dromaeosauridae; E, cf. *Richardoestesia gilmorei*; F, cf. *Richardoestesia isosceles*; G, cf. *Pectinodon*; and H, cf. *Troodon*. Qualitative characters: 1, posterior denticles apically oriented (that is, asymmetric denticles with a shorter apical side); 2, anterior denticles much smaller than posterior denticles; 3, posterior denticles rounded; 4, anterior denticles the same or slightly smaller than posterior denticles; 5, anterior denticles usually absent; 6, strong longitudinal ridges; 7, posterior denticles large and apically oriented; 8, posterior denticles are small and rounded; 9, anterior denticles are similar in size to posterior denticles or absent; 10, tall isosceles triangle shape; 11, posterior denticles very large and often rounded with apex of tooth frequently forming apical-most denticle; 12, posterior denticles are very large and apically hooked; and 13, anterior denticles are very large or absent. A, B, and H modified from Larson et al. 2010; C–F modified from Larson (2008); and G modified from Longrich (2008). Scale bars are 1 mm and correspond to images of crowns.

Eight qualitative morphotypes (forms identified by previous authors as “species”) were identified a priori (Fig. 2), and these were further separated into a total of 34 categories based on lithostratigraphic unit. Sample sizes for individual categories can be seen in Table 1. Each category, consisting of three specimens (at minimum) with usually at least four log-transformed measurements each, was compared to every other category in the statistical program JMP Version 5 [57]. The following tooth categories could not be analyzed due to a paucity of specimens with known measurements from a specific lithostratigraphic unit: Aguja and Javelina saurornitholestines [51], John Henry Member and lower Horseshoe Canyon dromaeosaurines (pers. obs.), Hell Creek and Lance cf. *Zapsalis* sp. ([53]; see Results section and Supporting Information (Text S1) for discussion of this taxon), and all specimens from the Foremost, Wapiti, and Scollard formations ([50]; pers. obs.). Because qualitative morphotypes of cf. *Paronychodon lacustris* from all formations typically lack denticles, only three variables could be analyzed. As such, these specimens are difficult to distinguish from those of other categories, and were not dealt with in this study. Similarly, measurements of the tooth taxon cf. Aves [50] are not analyzed because they lack denticle measurements and because specimens identified as such within a single formation may not be referable to a single taxon. As well, their assignment to Aves has been questioned [55].

**Table 1:** Individual tooth sample sizes of categories used in analyses by lithostratigraphic unit and qualitative morphotype.

		Qualitative morphotype							
		Sauronitholestinae	Dromaeosauridae	Dromaeosaurinae	cf.*Zapsalis*	cf.*Troodon*	cf.*Pectinodon*	cf.*R. gilmorei*	cf.*R. isosceles*
**Lithostratigraphic unit**	Milk River	191	8	24	11	–	–	225	67
	John Henry	–	–	–	–	–	–	8	4
	Oldman	20	–	6	–	–	–	4	10
	Dinosaur Park	112	–	76	23	36	3	16	24
	Judith River	–	–	–	–	7	–	–	–
	Two Medicine	10	–	–	–	–	–	–	–
	Aguja	–	–	–	–	–	–	–	–
	Horseshoe Canyon	31	–	13	–	36	–	9	–
	Lance	35	–	–	–	–	40	22	30
	Hell Creek	44	–	–	–	–	5	11	18

The subfamily name Saurornitholestinae was also used in lieu of the more commonly used designations of *Saurornitholestes langstoni* and subfamily Velociraptorinae. Previous authors [15], [17] have referred teeth in the Late Cretaceous of North America with pointed posterior denticles and smaller anterior denticles to the genus *Saurornitholestes*, and often to the species *Saurornitholestes langstoni*. The usage of this name should be restricted only to teeth from the Upper Campanian of the Dinosaur Park Formation and time-equivalent portions of the Oldman Formation of southern Alberta. The referral of isolated teeth to this taxon from equivalent units elsewhere should be limited to cases in which samples of several teeth may be compared to the Dinosaur Park teeth. Ideally, additional characters from other parts of the skeleton should be used. Traditionally referred to the dromaeosaurid subfamily Velociraptorinae, Longrich and Currie [58] erected the clade Saurornitholestinae for the eudromaeosaurians *Atrociraptor marshalli*, *Bambiraptor feinbergi*, and *Saurornitholestes langstoni*. It is to this subfamily that unnamed teeth from other sites in North America with a *Saurornitholestes*-like morphology should be referred, as it is not apparent that a similar tooth morphology is diagnostic to any higher group that includes *Velociraptor mongoliensis* [59], [60]. Outside of the context of Upper Cretaceous rocks of North America and Asia [21], [32], [33], [36], identifications of teeth with this morphology should conservatively be restricted to Eudromaeosauria indet. (sensu [58]) at the most specific taxonomic scale. This avoids inferring possibly incorrect biogeographic or taxonomic occurrences based on this likely symplesiomorphic morphology.

**Figure 3:**
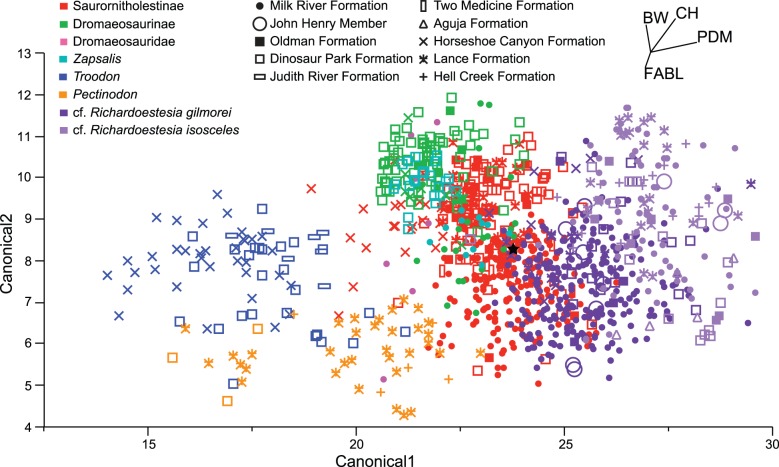
Canonical variate analysis of 1047 teeth in 32 categories distinguished by qualitative morphotype and chronostratigraphic unit. The black star indicates the centroid of the dataset from which the relative orientations of the biplot rays in the upper right were calculated. Canonical axes 1 and 2 indicate the first two axes of maximum discrimination in the dataset.

**Figure 4:**
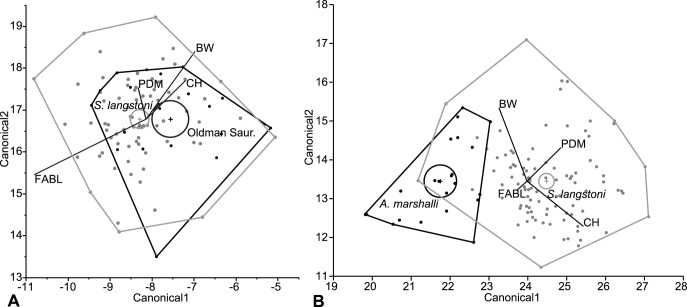
Example discriminant function analysis (DFA) canonical plots. A, *Saurornitholoestes langstoni* (Dinosaur Park; gray) vs. Oldman Saurornitholestinae (black), no discrimination of categories (hit ratio = 69.44%, *p* = 0.1363). B, *S. langstoni* (Dinosaur Park; gray) vs. *Atrociraptor marshalli* (Horseshoe Canyon; black), discrimination of categories (hit ratio = 94.215%, *p*<0.0001). Centroids (with 95% confidence interval) and associated convex hulls are labelled. Canonical axes 1 and 2 indicate the first two axes of maximum discrimination in the dataset.

Analyses included a CVA (Fig. 3) on 1047 directly comparable specimens in 32 categories as well as a DFA (Fig. 4) on the whole dataset analyzed in a pairwise fashion. CVA functions by calculating the multivariate mean (centroid) for each a priori category and maximizing the distance between these centroids [47]. Then individual points are classified according to their distance from the centroid and compared to the original a priori classification. New classifications that match a priori classifications are used to calculate a percent of correctly classified specimens, or hit ratio. DFA functions in the same way, but only uses two a priori groups [47]. Cross-validation, or re-running the discriminant analysis with each point removed to calculate a hit ratio, was run in the statistical program R [61] using the “lda” function in the MASS package [62] to ensure observed patterns were robust to missing specimens. Multivariate analyses of variance (MANOVA) were also conducted in JMP Version 5 [57] to determine the significance of the pairwise comparisons.

Following analysis, DFA hit ratios (the percentages of correctly identified specimens in the analysis) were then used to determine whether the categories are different enough to be considered distinct quantitative morphotypes (Fig. 4). Although Hammer and Harper [47] considered a 90 percent hit ratio the minimum for differentiating quantitative morphotypes using DFA, in this analysis, categories with hit ratios between 75 and 90 percent are here described as similar but considered distinct, and hit ratios between 75 and 85 percent were closely examined because of their quantitative similarity. This provided identifications of quantitative morphotypes most consistent with the findings of MANOVA. In addition to consideration of consistent patterns, DFA hit ratios lower than 90 percent were also accepted due to the nature of the material previously studied using DFA, that is, the same structures in different specimens [47] and not for serially homologous structures like the teeth used in the current analyses. In the few cases in which only three variables were analyzed, DFA and MANOVA gave consistent results. However, the hit ratios of DFA are often lower compared to analyses with a higher number of variables. Generally, categories with fewer than five specimens were observed to be less reliable in determining significance in MANOVA tests, and were avoided. Even in sample sizes between five and ten specimens, inconsistencies between DFA and MANOVA results are noted. This is probably because small samples do not display the range of variation likely present in the quantitative morphotype due to positional, ontogenetic, or individual variation.

Pairwise DFA analyses were utilized in addition to an overall CVA because, although similar patterns are reflected, the use of so many categories of specimens with so little in the way of quantified morphological variation does not provide a useful tool in discriminating at the specific level (Fig. 3). Similar difficulties in using CVA on theropod tooth datasets have been noted previously [45], [63]. When a large number of categories are analyzed, it is inevitable that some categories are going to be more similar than others, and large-scale differences (often corresponding to broader taxonomic levels) are the primary factor in clustering centroids, obscuring subtler differences in similar categories. It is only when these large scale differences are removed (when looking at similar categories) that subtler differences can be consistently observed.

Also, because DFA will find any consistent difference between categories, a test of the method was done by comparing the holotype specimen of the named taxa (if the holotype specimen possessed multiple measurable teeth in jaws) to referred isolated teeth from the same formation. As well, even if the holotype specimens consisted of single teeth or were otherwise not comparable using DFA, specimens were compared to evaluate similarity.

The current study did not account for effects of ontogeny in the sample prior to analysis. Although Buckley et al. [56] showed that significant differences between juvenile and adult tyrannosaur teeth can be observed, such a multifold increase in size through ontogeny is not reflected in the analyzed sample. Based on the overall sizes of the teeth with consistent denticle morphology, the range in size variation within a category is usually quite restricted. As well, correcting for size would eliminate a possible axis of discrimination that may be taxonomically controlled. While it is possible that some categories may be different ontogenetic stages of a single species separated by size alone, differences in denticle morphology usually preclude such arguments. No permits were required for the described study, which complied with all relevant regulations.

## Results

The CVA illustrates that quantitative analyses produce clustering similar to the qualitative morphotypes (Fig. 3). However, with a hit ratio (the percentage of specimens correctly identified according to their a priori identifications) of only 38.2 percent, the analysis is much too coarse to evaluate taxonomic distinction outside of a cursory visualization. This visualization is useful, however, in observing broad patterns of similarity amongst the disparate categories.

The pairwise comparisons provided a much clearer understanding of the relationships between categories. The DFA hit ratios combined with tests of significance from the MANOVA show that many of the qualitative morphotypes are distinct when they occur in different formations (Table 2; Fig. 4B). Conversely, when categories belonging to the same qualitative morphotype are from roughly time-equivalent formations, there is often no significant difference between them. For example, time-equivalent categories, such as much of the Oldman and Dinosaur Park formations, the Hell Creek and Lance formations, and the Milk River Formation and John Henry Member of the Straight Cliffs Formation (of Utah), are often indistinguishable from each other (Fig. 4A). Analyses conducted with only three variables often have decreased hit ratios. However, there seems to be little difference in results between analyses conducted with four and five variables, and large differences in quantity between categories seem to have little effect. Results from the cross-validation analyses were broadly similar to the results of the DFA (Table S2) except where noted.

**Table 2:** Hit ratios for each pairwise discriminant function analysis (DFA) of theropod tooth categories showing the percentage of correctly identified elements.

		Saurornitholestinae							Dromn.				*Zap.*		Dd.	*Troodon*			*Pectin.*			cf. *R. gilmorei*							cf. *R. isosceles*						
		M	T	O	D	Ho	L	He	M	O	D	Ho	M	D	M	D	J	Ho	D	L	He	M	JH	O	D	Ho	L	He	M	JH	O	D	A	L	He
Saurornitholestinae	Milk River	X	93	91	87	99	94	77*	92	100	99	99	96	100	98	100	100	100	100	100	100	82	93	89	89	95	87	79*	98	100	100	100	98	100	99
	Two Medicine (*B. feinbergi*)		X	87	92	100	97	91*	85	100	96	96	95	100	100	100	100	100	100	100	100	92	100	88	97	100	100	90*	99	100	100	100	100	100	96
	Oldman			X	**69**	100	83	80*	84	92	97	91	90	98	100	100	100	100	100	98	96	96	93	92	90	87	91	90*	99	100	100	100	100	100	97
	Dinosaur Park (*S. langstoni*)				X	95	80	69*	86	93	94	87	95	93	95	98	99	99	100	98	98	95	93	93	86	89	87	84*	99	100	100	98	100	99	98
	Horseshoe Canyon (*A. marshalli*)					X	94	86*	85	96	90	83	97	96	87	96	100	98	100	100	100	100	100	100	100	100	100	93*	99	100	100	100	100	100	100
	Lance						X	70*	75	91	89	83	95	100	91	100	100	100	100	100	100	99	100	100	94	92	95	78*	99	100	100	100	100	100	100
	Hell Creek							X	76*	88*	87*	91*	91*	93*	96*	100*	100*	100*	100*	100*	100*	88*	87*	78*	80*	76*	77*	67*	94*	100*	96*	94*	98*	95*	92*
Dromaeosaurinae	Milk River								X	90	90	92	80	98	84	100	100	100	100	100	100	96	100	100	96	94	92	76*	99	100	100	100	100	100	100
	Oldman									X	**67**	95	100	100	100	100	100	100	100	100	100	100	100	100	100	100	100	100*	99	100	100	100	100	100	100
	Dinosaur Park (*D. albertensis*)										X	77	95	94	95	100	100	100	100	100	100	100	100	99	100	96	99	92*	99	100	100	100	100	100	100
	Horseshoe Canyon											X	88	94	**86**	100	100	100	100	100	100	100	100	100	97	96	96	96*	99	100	100	100	100	100	100
cf. *Zapsalis*	Milk River												X	100	100	100	100	100	100	100	100	96	100	100	100	95	100	95*	99	100	100	100	100	100	100
	Dinosaur Park (cf. *Z. abradens*)													X	94	100	100	100	100	100	100	100	100	100	100	100	100	100*	100	100	100	100	100	100	100
Dromaeosauridae	Milk River														X	100	100	100	100	100	100	100	100	100	100	95	100	100*	99	100	100	100	100	100	100
cf. *Troodon*	Dinosaur Park (*T. inequalis*)															X	**76**	84	100	100	93	100	100	100	100	100	100	100*	100	100	100	100	100	100	100
	Judith River (*T. formosus*)																X	97	100	100	100	100	100	100	100	100	100	100*	100	100	100	100	100	100	100
	Horseshoe Canyon																	X	97	99	95	100	100	100	100	100	100	100*	100	100	100	100	100	100	100
cf. *Pectinodon*	Dinosaur Park																		X	**81**	**100**	100	100	100	100	100	100	100*	100	100	100	100	100	100	100
	Lance (*P. bakkeri*)																			X	**54**	100	100	100	100	100	100	100*	100	100	100	100	100	100	100
	Hell Creek																				X	100	100	100	100	100	100	100*	100	100	100	100	100	100	100
cf. *R. gilmorei*	Milk River																					X	**59**	75	80	91	88	87*	90	96	91	90	89	95	93
	John Henry																						X	100	**72**	84	95	89*	90	100	95	100	100	91	96
	Oldman																							X	85	94	100	76*	85	100	100	93	100	93	79
	Dinosaur Park (*R. gilmorei*)																								X	**69**	**63**	75*	84	100	88	86	96	87	85
	Horseshoe Canyon																									X	**80**	68*	89	93	82	91	100	89	86
	Lance																										X	72*	89	94	88	86	100	89	94
	Hell Creek																											X	93*	100*	100*	97*	100*	95*	93*
cf. *R. isosceles*	Milk River																												X	**80**	**63**	61	74	65	**63**
	John Henry																													X	87	93	**100**	89	95
	Oldman																														X	**53**	**69**	83	**79**
	Dinosaur Park																															X	**71**	79	80
	Aguja (*Richardoestesia isosceles*)																																X	97	100
	Lance																																	X	**62**
	Hell Creek																																		X

Bolded numbers indicate a non-significant differences in MANOVA; * indicates analyses with only three variables. [full page width].

**Figure 5:**
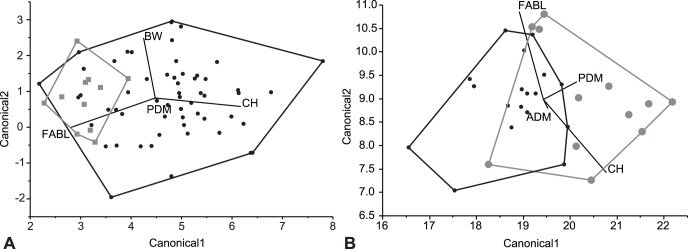
Discriminant function analysis of type specimens versus referred isolated teeth. A, *Dromaeosaurus albertensis*. B, *Atrociraptor marshalli*. Gray indicates holotype specimen teeth, black indicates referred isolated teeth. Associated convex hulls are marked. Canonical axes 1 and 2 indicate the first two axes of maximum discrimination in the dataset.

Three taxa are known from teeth associated with holotype specimens that include skeletal material and can be compared directly with isolated teeth referred to these taxa. The teeth of the holotype of *Saurornitholestes langstoni* compared to isolated teeth also from the Dinosaur Park Formation are not significantly different (hit ratio of 72%; *p* = 0.6736). However, teeth referred to *Atrociraptor marshalli* from the Horseshoe Canyon Formation were statistically distinct from the holotype specimen of this species (also from the Horseshoe Canyon Formation) using ADM, CH, FABL, and PDM (hit ratio of 77%; *p* = 0.0112; Fig. 5B). Similarly, the maxillary and dentary teeth of the holotype of *Dromaeosaurus albertensis* (from the Dinosaur Park Formation) are distinguishable from isolated teeth from the Dinosaur Park Formation that have been referred to this taxon (hit ratio of 76%; *p* = 0.0037; Fig. 5A). Although these results indicate that the holotype specimens of *Atrociraptor* and *Dromaeosaurus* are distinguishable from referred isolated teeth from within the same formation, the hit ratios are quite low–just above the low cut-off used in this study for a few cases. This seeming distinction may be due to the holotypes not preserving the full ranges of variation for the taxa. Indeed, in both of these exceptions, there are some referred specimens that group quite closely with holotype teeth, while others preserve morphology seemingly distinct from the holotype (Fig. 5). In comparisons of teeth of all three holotypes to the same qualitative morphotypes from different formations, hit ratios are all over 85 percent. This indicates that the holotype specimen teeth are, at least, more similar to referred isolated teeth from the same formation than they are to any other category. The holotype specimens of other species are inadequate for these kinds of analyses because they are either single teeth (*Pectinodon bakkeri*, *Richardoestesia isosceles*, *Troodon formosus*, *Zapsalis abradens*), or the teeth of the type specimen are germ teeth (*Richardoestesia gilmorei*) that are incompletely erupted. However, measurements for holotype teeth of *Richardoestesia isosceles*, *Troodon formosus*, and *Zapsalis abradens* are consistent with measurements from equivalent formations, and similarities between the germ teeth of *Richardoestesia gilmorei* and referred teeth from the Dinosaur Park Formation have been noted previously [14], [50].

### Recognized Quantitative Morphotypes

#### Saurornitholestinae

The Milk River Formation saurornitholestine is significantly different from all other theropod tooth categories in MANOVA and DFA, and is regarded as a distinct quantitative morphotype. When compared, only five categories have less than 90% hit ratios: *Saurornitholestes langstoni*, and four cf. *Richardoestesia* taxa. Of these, only one (the Milk River cf. *Richardoestesia gilmorei*) is lower than 85%. The similarity between the Milk River saurornitholestine and cf. *Richardoestesia gilmorei* teeth has been noted in the past [15]. However, the significantly different means calculated in MANOVA and differing denticle morphology suggests the similarity likely represents convergence in tooth morphology in two distinct taxa.

Teeth from the type specimen of *Bambiraptor feinbergi* are distinct from all other categories in both DFA and MANOVA analyses and are regarded as a distinct quantitative morphotype. Three categories have hit ratios of less than 90% (Milk River Dromaeosaurinae, Oldman Saurornitholestinae, and Oldman cf. *R. gilmorei*), but all ratios are higher than 85%. This result may be questionable, however, based on the somewhat ambiguous results of the other holotype comparisons and the immature nature of the holotype of *Bambiraptor feinbergi* [64]. Additionally, the cross-validation analysis indicates great similarity with saurornitholestine teeth from the Oldman formation, possibly indicating multiple morphotypes present in that formation.

The Dinosaur Park *Saurornitholestes langstoni* category is indistinguishable from the Oldman Saurornitholestinae category in DFA and MANOVA (Fig. 4A). Saurornitholestine teeth from the Oldman and Dinosaur Park formations are here regarded as a single quantitative morphotype: *Saurornitholestes langstoni*. Both the Oldman and Dinosaur Park categories are similar to (hit ratio less than 90%) the Horseshoe Canyon cf. *Richardoestesia gilmorei*, the Lance saurornitholestine, and the Milk River dromaeosaurine. The Dinosaur Park category is similar to the upper Horseshoe Canyon dromaeosaurine, and the Dinosaur Park and Lance cf. *Richardoestesia gilmorei*. Again, only the Lance category has hit ratios lower than 85% for both the Oldman and Dinosaur Park and saurornitholestine categories. This suggests a close similarity between the Lance and *Saurornitholestes langstoni* morphotypes. Although specimens from the Foremost Formation do not have high enough sample sizes to yield consistent results, it is suspected that they would be similar to other Belly River Group (Oldman and Dinosaur Park formations) teeth, but distinct morphotypes would not be unexpected due to the lower stratigraphic position of these specimens.

Although measurements from the teeth of the holotype of *Atrociraptor marshalli* are significantly different from those of the referred teeth from the same formation, the hit ratio of 77% is quite low. A similarly low hit ratio (77%) occurred when comparing cf. *Atrociraptor marshalli* teeth from the upper to the lower Horseshoe Canyon Formation, but in this case, the difference was not significant in MANOVA (*p* = 0.5625). Although it is not below the 75% cut-off, the relatively low hit ratio, the morphological similarity of the denticles, and the stratigraphic provenance of the specimens supports the inclusion of all referred teeth from the Horseshoe Canyon Formation as *Atrociraptor marshalli*. It is hypothesized that the apparent significant difference may indicate the isolated tooth sample does not possess specimens from the full range of *Atrociraptor* morphology. Greater sampling of this category may resolve this problem. The teeth in the *Atrociraptor marshalli* category are distinct from those of the *Saurornitholestes langstoni* category (Fig. 4B). The only quantitatively similar categories to *Atrociraptor marshalli* are the Milk River and Horseshoe Canyon dromaeosaurines and the Milk River dromaeosaurid; none of these pairings have hit ratios lower than 85%.

The Lance saurornitholestine (identified as such here because of the dissimilarity of size between anterior and posterior denticles) matches the Hell Creek saurornitholestine in both DFA and MANOVA, although only three variables were analyzed. Previous descriptions of Lancian dromaeosaurids [53], [55] have differed in terms of the number of taxa recognized (one in the former, three in the latter). Based on the similarity of available published measurements and descriptions, dromaeosaurids from the Hell Creek and Lance formations are here analyzed with one category per formation (although see the subsequent discussion of *Zapsalis*). Both categories in the current analyses are distinct from all other categories in both DFA and MANOVA and are regarded as a distinct morphotype. As mentioned earlier, the three variables analyzed for the Hell Creek saurornitholestine DFA produce lower hit ratios than those in the quantitatively indistinguishable Lance specimens in which four variables were analyzed. Additional categories similar to the Lance saurornitholestine include the Milk River (hit ratio 75%) and upper Horseshoe Canyon (hit ratio 83%) dromaeosaurine, and *Dromaeosaurus albertensis* (hit ratio 89%). However, all three of these ratios increase when five variables are analyzed, to 84%, 95%, and 100%, respectively. Cross-validation analyses also indicated a close similarity with Milk River dromaeosaurines (Table S2).

**Figure 6:**
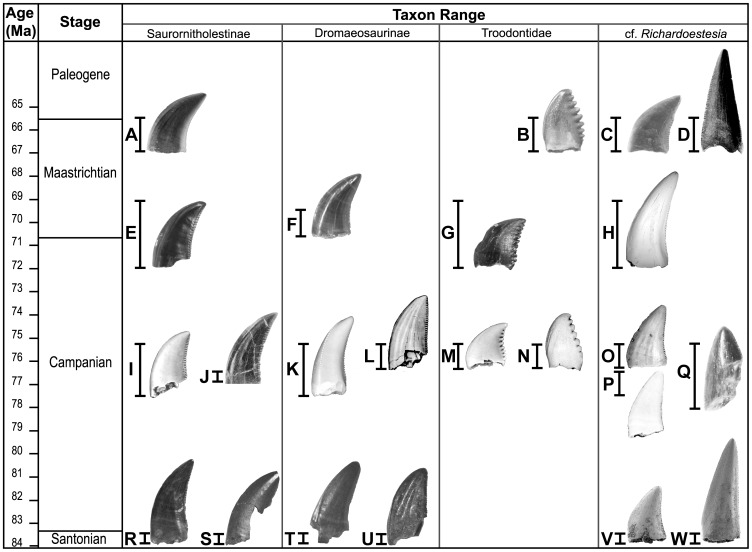
Summary of quantitative morphotypes showing their stratigraphic ages. Each tooth icon likely represents a distinct taxon with the indicated known range based on formation as observed in this study. A, Lancian Saurornitholestinae gen. et sp., UCMP 187036 (reversed); B, *Pectinodon bakkeri*; C, Lancian cf. *Richardoestesia gilmorei*, UCMP 120255 (reversed); D, Lancian cf. *Richardoestesia isosceles*, UCMP 187175 (reversed); E, *Atrociraptor marshalli*, TMP 2000.045.0035; F, Horseshoe Canyon Dromaeosaurinae gen. et sp., TMP 1999.050.0116 (reversed); G, Horseshoe Canyon cf. *Troodon* sp., TMP 2000.045.0024 (reversed); H, Horseshoe Canyon cf. *R. gilmorei*, TMP 2003.015.0002; I, *Saurornitholestes langstoni*, TMP 1995.147.0026; J, *Bambiraptor feinbergi*, AMNH FR 30556; K, *Dromaeosaurus albertensis*, TMP 1986.130.0211; L, *Zapsalis abradens*, TMP 1987.050.0008; M, *Troodon formosus*, TMP 1995.147.0025; N, Dinosaur Park cf. *Pectinodon* sp., TMP 2000.021.0001; O, *Richardoestesa gilmorei*, TMP 2000.019.0004; P, Oldman cf. *R. gilmorei*, 1987.080.0035; Q, *Richardoestesia isosceles*, LSUMGS 489∶6238 (reversed); R, Milk River Saurornitholestinae gen. et sp., UALVP 50531 (reversed); S, Milk River Dromaeosauridae gen. et sp., UALVP 48365 (reversed); T, Milk River Dromaeosaurinae gen. et sp., UALVP 49571; U, Milk River cf. *Zapsalis* sp., UALVP 49582; V, Aquilan cf. *Richardoestesia gilmorei*, UALVP 48157 (reversed); and W, Aquilan cf. *Richardoestesia isosceles*, UALVP 48279 (reversed). B modified from Longrich (2008); H modified from Larson et al. (2010); J modified from Burnham (2004); P modified from Sankey et al. (2002); Q modified from Sankey (2001); R–W modified from Larson (2008). Teeth scaled to matching FABL. [full page width].

The Milk River dromaeosaurid [46] has hit ratios of at least 84% (the only pairing below 85%) when compared to other categories. All MANOVA tests for significance indicate a significant difference in the multivariate means, except for one category (upper Horseshoe Canyon dromaeosaurine), which also showed great similarity in the cross-validation analysis (Table S2). The fact that the Milk River dromaeosaurid and upper Horseshoe Canyon dromaeosaurine categories lack a significant difference in variables is likely an artefact of the small sample size (11 and eight specimens, respectively), and would likely resolve with greater sampling. As well, the denticle shape, stratigraphic provenance, and DFA of five variables resulting in a 95% hit ratio all do not support referral to the same quantitative morphotype. The morphology of the denticles of these teeth does not strongly indicate placement in either the Saurornitholestinae or Dromaeosaurinae; however, the morphology was united graphically with Saurornitholestinae in Figure 6 for convenience.

#### Dromaeosaurinae

Teeth of the Milk River dromaeosaurine, identified as such because of the shape and the lack of relative size differences between anterior and posterior denticles, are distinct from all other categories in both DFA and MANOVA analyses. In addition to those similarities already mentioned, the only other similarity is with the Milk River cf. *Zapsalis* sp. (hit ratio 80%, cross-validated hit ratio 77%). While it is possible that these two categories represent two morphologies of teeth within an individual taxon, the fact that their distinctiveness is supported by the current analyses supports their tentative separation as different quantitative morphotypes.

*Dromaeosaurus albertensis* type material is shown in DFA to be distinguishable from referred teeth from the Dinosaur Park Formation (hit ratio of 78%). As well, MANOVA results in *p* = 0.0004, a significant difference in multivariate means. This may indicate that the type material does not adequately represent the variability present in the species as seen in the isolated teeth. In DFA and MANOVA, this category is also shown to be identical to that from the Oldman Formation. These results support the referral of the Oldman and Dinosaur Park categories to *Dromaeosaurus albertensis*. This corroborates the occurrence of the qualitatively characteristic twisted anterior carina of the species to the exclusion of all other taxa.

The upper Horseshoe Canyon dromaeosaurine category represents a distinct quantitative morphotype in all comparisons, with similarities as noted in previous paragraphs. Although specimens measured for this study were too few (n = 3) to provide reliable results, analyses performed indicate that the lower Horseshoe Canyon dromaeosaurine is distinct from the upper Horseshoe Canyon dromaeosaurine (hit ratio of 94%). When the lower category is compared to Oldman and Dinosaur Park dromaeosaurine teeth, hit ratios of 82% and 67% respectively are recorded. Therefore, quantitatively, the lower Horseshoe Canyon teeth seem indistinguishable from those of *Dromaeosaurus albertensis* although lower Horseshoe Canyon teeth lack the distinctive twisted anterior carinae that characterize these teeth. It is tentatively suspected that with greater sampling, the dromaeosaurine teeth of the lower Horseshoe Canyon Formation will be supported as distinct from both the upper Horseshoe Canyon morphotype and *Dromaeosaurus albertensis*.

Teeth referred to *Zapsalis abradens* from the Dinosaur Park Formation correspond closely to the measurements of the type specimen from the Judith River Formation described by Cope [65] (see Text S1). These specimens are characterized by rounded dromaeosaurine-like denticles, a straight posterior carina, and pronounced longitudinal ridges resembling those of *Paronychodon lacustris*. *Zapsalis abradens*, in the current study, is regarded as valid because the distinct morphology is absent in the type specimen of *Dromaeosaurus albertensis*. These teeth correspond to those referred to as *Dromaeosaurus* Type A by Sankey et al. [50]. *Zapsalis abradens* is distinct from all of the other categories in both DFA and MANOVA analyses, with hit ratios of at least 93% in all pairings.

The teeth of the Milk River cf. *Zapsalis* sp. (which possess ridges like those of *Z. abradens*) is differentiated from every other category in both analyses and is supported as a distinct quantitative morphotype. The category was similar, in addition to those similarities already noted, to the upper Horseshoe Canyon dromaeosaurine, but the hit ratio (88%) does not indicate a particularly close similarity. Categories referable to the cf. *Zapsalis* qualitative morphotype also may rarely occur in the Hell Creek and Lance formations [53]. However, the available sample size of these teeth is not great enough to provide reliable results in the analyses.

#### Troodontidae

The holotype tooth of *Troodon formosus* came from the Judith River Formation in Montana [66]. Referred *Troodon formosus* teeth from this formation are shown to be indistinguishable from *Troodon* teeth from the Dinosaur Park Formation of Alberta in MANOVA (*p* = 0.0556). DFA had a hit ratio of 76%, close to the 75% cut-off. Given the close proximity of the geographic and stratigraphic provenance of these two categories, and the results of DFA and MANOVA analyses, these two categories are regarded as the same quantitative morphotype: *Troodon formosus*. They are not similar to any other category of teeth in the DFA; however, in the cross-validation analysis, the Dinosaur Park morphotype was more similar to the Horseshoe Canyon morphotype than to that from the Judith River (Table S2). Measurements of troodontids from the Oldman Formation were not available, although specimens are known to exist [67].

Troodontid teeth from the Horseshoe Canyon Formation are distinct from the Dinosaur Park Formation teeth in DFA and MANOVA (hit ratio 84%; *p*<0.0001). As well, no similarity is seen in comparisons between the Horseshoe Canyon and Judith River *Troodon* (hit ratio 97%; *p*<0.0001). Given these results, these teeth are regarded as distinct quantitative morphotypes even though Currie [68] found no basis for separating the recovered skeletal material found in the Dinosaur Park and Horseshoe Canyon formations as distinct species.

*Pectinodon bakkeri* was described on the basis of dental material from the Lance Formation [69]. It was found to be a valid taxon by Longrich [55] based on qualitative characters. Both DFA and MANOVA in the current study show discrimination of *P. bakkeri* teeth to the exclusion of every other category except those of cf. *Pectinodon* from the Hell Creek Formation, indicating that these categories are the same quantitative morphotype. Teeth referable to *Pectinodon* have also been documented from the Dinosaur Park Formation [50] although in low quantities. These teeth were referred to “cf. Troodontidae gen. et sp. indet. A” by Sankey et al. [50], but based on the rounded shape of the denticles and distinct longitudinal ridges, these teeth are likely referable to cf. *Pectinodon*. DFA shows these teeth to be distinct from all other categories of teeth analyzed, including hit ratios of 81% and 100%, respectively, for the Lance and Hell Creek *Pectinodon* categories. MANOVA indicates that the multivariate mean of the Dinosaur Park category is not significantly different from either of the Lancian categories (*p* = 0.0700 for the Lance Formation and *p* = 0.0842 for the Lance Formation), but this is likely due to small sample size. The Dinosaur Park cf. *Pectinodon* is regarded as a distinct quantitative morphotype.

#### Coelurosauria incertae sedis: the *Richardoestesia* complex

Teeth referred to the genus *Richardoestesia* present a taxonomic problem in the fossil record of North America. This problem is partly due to the close morphological similarity of teeth referred to the genus, despite great disparity of ages and locations. Given the results of both of the analyses, a tentative identification of different quantitative morphotypes referred to this genus can be reached.

The holotype of *Richardoestesia gilmorei*, because of its possession of only germ teeth, does not facilitate comparison with measurements of shed teeth. Teeth are characterized by their rounded, small denticles and posteriorly curved crowns [14]. Shed teeth from several North American formations have been referred to the taxon [15].

The holotype of *R. isosceles*, known only from shed tooth crowns, is known from the Aguja Formation of Texas, although specimens from more northern Santonian−Maastrichtian units have also been referred to this taxon [17]. The species is characterized by relatively tall, straight crowns. However, Rauhut [32] argued that the species was not sufficiently diagnosed and regarded *R. isosceles* as a nomen dubium. Longrich [55], based on the anterior tooth morphology of the type specimen of *R. gilmorei*, regarded *R. isosceles* as a subjective junior synonym of *R. gilmorei*. Here, the qualitative morphotypes of cf. *R. gilmorei* or cf. *R. isosceles* are analyzed to determine what quantitative morphotypes are present. The use of these names is not to imply referral of these teeth to either species, but simply to denote that cf. *R. gilmorei* teeth are curved and often short, and cf. *R. isosceles* teeth are straight and tall.

The Milk River cf. *R. gilmorei* is distinct in both DFA and MANOVA from all other categories analyzed except for the cf. *R. gilmorei* from the time-equivalent John Henry Member of the Straight Cliffs Formation of Utah [70]. These categories together are regarded as a distinct quantitative morphotype. Except as previously mentioned, the only categories similar to the Milk River cf. *R. gilmorei* are the Oldman, Dinosaur Park, and Lance cf. *R. gilmorei*, and the Aguja *R. isosceles*. Only the Oldman and Dinosaur Park cf. *R. gilmorei* hit ratios are lower than 85%. The John Henry cf. *R. gilmorei* is not significantly different from the Dinosaur Park *R. gilmorei* and has a hit ratio of 72% (although this improves to 100% when five variables were analyzed). As well, although significant in MANOVA, a hit ratio of 84% indicates a similarity to the Horseshoe Canyon cf. *R. gilmorei*.

Oldman cf. *R. gilmorei* teeth are distinct from all other categories in both DFA and MANOVA. The teeth are similar to those of the Dinosaur Park *R. gilmorei* and the Milk River and Lance *R. isosceles* as well as those previously mentioned. However, only the teeth of the Milk River cf. *R. gilmorei* and the Hell Creek *R. isosceles* have hit ratios lower than 85%. This category is tentatively regarded as a distinct quantitative morphotype until more specimens are available.

With four variables analyzed in DFA and MANOVA, the Dinosaur Park *R. gilmorei* is only indistinguishable from the Horseshoe Canyon and Lance cf. *R. gilmorei*. However, both analyses find the Dinosaur Park category to be distinct when five variables are analyzed with hit ratios of 100% and 94% and *p*-values of 0.0130 and 0.0314, respectively. When compared to the Milk River cf. *R. isosceles*, the hit ratio is 84%, and other similar categories not previously mentioned are the Oldman, Dinosaur Park, Lance, and Hell Creek cf. *R. isosceles* (hit ratios ranging from 85–88%). These results illustrate that even though they are similar morphologically, there exists a distinct difference between the two qualitative morphotypes of *Richardoestesia*. Therefore, in the current study, both species names are retained as distinct. *R. gilmorei* (restricted in occurrence in the current study to the Dinosaur Park Formation) is here regarded as a distinct quantitative morphotype from similar specimens in any other formation analyzed. This conclusion is somewhat less certain when the cross-validation analysis is taken into account (Table S2), which indicates that *R. gilmorei* and *R. isosceles* may not be different within the Oldman and Dinosaur Park formations. This may be the result of misidentification, and closer quantitative analysis of these morphotypes is necessary.

Compared to the remaining teeth in the current study, the Lance cf. *R. gilmorei* is distinct in DFA and MANOVA. However, similarities (DFA hit ratio<90%) are found with the Milk River, Oldman, Dinosaur Park, and Lance cf. *R. isosceles*, but none of the hit ratios are less than 85%. The Hell Creek cf. *R. gilmorei* is only analyzed using three variables, and is significantly different in MANOVA, but has a hit ratio of 72%. These two categories are tentatively regarded as the same quantitative morphotype. It is noteworthy that both of these categories, when compared to the cf. *R. isosceles* categories of the same formation, have DFA hit ratios ranging from 89−95% and are always significantly different in MANOVA.

The Milk River cf. *R. isosceles* presents an interesting problem in the current study. In addition to the similarities already noted, this category is not significantly different from the John Henry, Oldman, and Hell Creek cf. *R. isosceles*. As well, DFA hit ratios are below the 75% cut-off in the Oldman, Dinosaur Park, Aguja, Lance, and Hell Creek cf. *R. isosceles* teeth. The Dinosaur Park, Aguja, and Lance categories are significantly different from the Milk River category in MANOVA but only have hit ratios ranging from 61−74%, a situation difficult to interpret. The comparison with the John Henry category indicates that it is distinct from the Milk River category (hit ratio 80%), so the non-significant difference may be due to the small sample size of the John Henry category (n = 3). The John Henry category is additionally similar only to the Oldman and Lance categories, but neither hit ratio is less than 85%. As well, the John Henry category is not significantly different from the Aguja category, but this may be due to small sample size. Similarly, the cross-validation shows great similarity to both Oldman and Aguja morphotypes (Table S2). The John Henry cf. *R. isosceles* is here regarded as a distinct quantitative morphotype, but whether the Milk River cf. *R. isosceles* is part of this morphotype, its own quantitative morphotype, or part of another cf. *R. isosceles* morphotype is not known definitively.

The holotype of *Richardoestesia isosceles* is a partial tooth from the Aguja Formation of Texas [17]. Comparisons of teeth of this qualitative morphotype from the Aguja Formation yield results in which they appear to be distinct from all other categories except for the Oldman and Dinosaur Park cf. *R. isosceles*. The Oldman and Dinosaur Park teeth are also not distinct from each other in both DFA and MANOVA, and are similar to the Lance and Hell Creek categories (with hit ratios ranging from 79–83%). MANOVA indicates no significant difference between the Oldman and Hell Creek cf. *R. isosceles* (*p* = 0.0679), but this is regarded as an artefact of sample size. Here, the Oldman, Dinosaur Park, and Aguja categories are regarded as belonging to the same quantitative morphotype: *Richardoestesia isosceles*. At this time, it is advisable to restrict specimens referred to this species to those within time equivalent units in western North America. Referral of specimens of cf. *R. isosceles* from the Horseshoe Canyon, Lance, and Hell Creek formations is not supported, and referral of specimens from the Milk River Formation is equivocal (although for the John Henry Member of the Straight Cliffs Formation, referral is not supported). Although no specimens from the Frenchman and Scollard formations were analyzed, their stratigraphic positions suggest that specimens from these formations are probably not referable to this species.

As previously mentioned, there is good discrimination and significant differences between all other categories and both the Lance and Hell Creek cf. *R. isosceles*. When comparing the Lance and Hell Creek categories, a hit ratio of only 62% and a *p*-value of 0.3749 are calculated. These two categories are regarded as the same quantitative morphotype.

## Discussion and Conclusions

Using the results of the pairwise DFA, stratigraphic ranges of quantitative morphotypes can be figured (Fig. 6) and robust minimum estimates of diversity can be tabulated (Table 3). Each distinguishable quantitative morphotype found in the analyses likely represents a distinct taxon [47], bringing the minimum number of small theropod taxa in the last 19 million years of the Cretaceous of western North America to 23 (not including *Paronychodon lacustris*, which may be a valid taxon, and birds). According to these results, known diversity of small theropods in western North America shows a pattern that is highest in the late Campanian (76.5–74.8 Ma) and reduced prior to the end-Cretaceous mass-extinction, a pattern that has been suggested in other dinosaur taxa by previous authors [1], [5], [7], [71]. Based on the results of this study, it is possible that this reduction starts as early as the latest Campanian. However, diversity in the upper Campanian to lower Maastrichtian Horseshoe Canyon Formation may be underestimated due to poor sampling in other contemporaneous formations, particularly those from lower latitudes that may have had higher diversities [72].

**Table 3:** Table. 3. Taxonomic identifications of small theropods with teeth in the formations used in this study based on holotype material, referred skeletal material, and the results of the current study.

Lithostratigraphic Unit	Taxa (Quantitative morphotypes)
Milk River Formation	Milk River Saurornitholestinae gen. et sp.
	Milk River Dromaeosauridae gen. et sp.
	Milk River Dromaeosaurinae gen. et sp.
	Milk River cf. *Zapsalis* sp.
	Aquilan cf. *Richardoestesia gilmorei*
	?Aquilan cf. *Richardoestesia isosceles*
John Henry Member	Aquilan cf. *Richardoestesia gilmorei*
	Aquilan cf. *Richardoestesia isosceles*
	Saurornitholestinae indet.
	Dromaeosaurinae indet.
Oldman Formation	*Saurornitholestes langstoni*
	*Dromaeosaurus albertensis*
	Oldman cf. *Richardoestesia gilmorei*
	*Richardoestesia isosceles*
	Troodontidae indet.
Dinosaur Park Formation	*Saurornitholestes langstoni*
	*Dromaeosaurus albertensis*
	*Zapsalis abradens*
	*Troodon formosus*
	Dinosaur Park cf. *Pectinodon* sp.
	*Richardoestesia gilmorei*
	*Richardoestesia isosceles*
Two Medicine Formation	*Bambiraptor feinbergi*
	no isolated teeth from this unit analyzed
Judith River Formation	*Zapsalis abradens*
	*Troodon formosus*
	no other isolated teeth from this unit analyzed
Aguja Formation	*Richardoestesia isosceles*
	Saurornitholestinae indet.
Horseshoe Canyon Fm.	*Atrociraptor marshalli*
	Horseshoe Canyon Dromaeosaurinae gen. et sp.
	Horseshoe Canyon cf. *Troodon* sp.
	Horseshoe Canyon cf. *R. gilmorei*
Lance Formation	Lancian Saurornitholestinae gen. et sp.
	*Pectinodon bakkeri*
	Lancian cf. *Richardoestesia gilmorei*
	Lancian cf. *Richardoestesia isosceles*
Hell Creek Formation	Lancian Saurornitholestinae gen. et sp.
	*Pectinodon bakkeri*
	Lancian cf. *Richardoestesia gilmorei*
	Lancian cf. *Richardoestesia isosceles*

Teeth that can be identified as cf. *Paronychodon lacustris* (sensu Currie et al., 1990) occur in all formations.

These results show that quantitative identification of small theropod teeth is possible, even when comparable skeletal material is lacking. The method employed was consistent in all of the formations tested, and may be useful globally in other geographic locations for different time periods, particularly those with poor skeletal representation of specimens. Resolution was especially high between teeth of taxa from different families, so future quantitative comparisons need only be made between samples of teeth not readily separated by easily observed qualitative characters. Use of this method in other poorly known fossil groups with specimens of limited qualitative diagnostic potential is another avenue for future research. It is useful to keep in mind, however, that these methods are dependent on having sufficiently large sample sizes.

The holotype and referred material from time-equivalent formations of *Atrociraptor marshalli*, *Dromaeosaurus albertensis*, and *Saurornitholestes langstoni* have low hit ratios, which provide good support for the usefulness of this methodology. However, further research is necessary to evaluate this method using multiple specimens with teeth in jaws. Taxa for which known in situ teeth exist seem to provide more robust analyses in closely related taxa, as illustrated by the well-resolved dromaeosaurids and sometimes poorly-resolved *Richardoestesia* taxa. Small sample sizes of less than ten teeth for some categories sometimes yield inconsistent results that are more difficult to interpret. As well, more closely related taxa appear to have similar teeth that are difficult to distinguish from each other, potentially limiting these analyses for use with closely related taxa with essentially identical dentitions.

The results of this study suggest that *Richardoestesia gilmorei* is only definitively known from the Dinosaur Park Formation, and it does not seem to include the contemporaneous *R. isosceles*. *Dromaeosaurus albertensis* and *Saurornitholestes langstoni* are here regarded as occurring only in the Oldman and Dinosaur Park formations whereas *Troodon formosus* (sensu stricto) is only definitively known from the Dinosaur Park and Judith River formations. These occurrences, of course, must be evaluated as new material becomes available.

While other tooth morphotypes may be quantitatively distinct and probably represent different taxa, naming of such taxa would be premature until more complete specimens with skeletal elements are known. Tooth characters such as denticle size, crown size, and crown shape have been shown to vary within taxa and may exhibit morphologies convergent with other taxa [17], [22], [32], [45], [73], limiting the usefulness of these characters for diagnosis. Similarly, the difficulty of distinguishing quantitative morphotypes assigned to *Richardoestesia* would likely be alleviated by the discovery of specimens with teeth in jaws, allowing researchers to confidently identify teeth to their appropriate qualitative morphotype. Although there is some broad agreement in these quantitative results, the difficulty in distinguishing morphotypes may, in some cases, be attributable to incorrect a priori identifications.

Finally, it is worth emphasizing that the study also suggests it is unlikely that taxa can be referred to a known species if there are significant temporal or geographic differences between specimens, and it is unwise to assign existing species names to temporally or geographically isolated material without quantifying similarities. These results indicate that small theropod diversity in the Late Cretaceous of western North America is much greater than presently recognized, and suggests that theropod species turnover is roughly equivalent to that in other dinosaurian lineages [42], [43]. Subtle qualitative differences can be attributed to differences within populations of single species [15], but due to consistent stratigraphically separated morphologies and a near absence of well-preserved small theropod taxa, it is better to look at this variation in isolation until more complete specimens are found and compared. It is clear from this study that even within relatively small stratigraphic and geographic ranges in the Cretaceous of western North America, the teeth of related small theropods can be distinguished quantitatively, even if they share diagnostic, qualitative characters. On a local scale, the identification of isolated teeth can be undertaken with confidence, especially if there is associated diagnostic skeletal material, and quantitative and qualitative differences can provide strong evidence of ecological, geographic, stratigraphic, and/or taxonomic differences. The interpretation of distinct qualitatively similar tooth morphologies from sites that are geographically or temporally distant may be biologically significant. However, they must be supported by other lines of evidence (such as quantitative analysis or analysis of other parts of the skeleton, or associated flora and fauna) before they are considered significant.

## Supporting Information

Table S1 **Measurements for small theropod teeth with source data reference.** Abbreviations as in Figure 1. *, holotype specimen.
(XLS)

Table S2 **Pairwise cross-validation discriminant function analysis (DFA) hit ratios.** White cells indicate hit ratios over 90%; light gray indicates analyses with three variables with hit ratios between 75% and 90%; middle gray indicates analyses with four variables between 75% and 90% and analyses with three variables less than 75%; and dark gray indicates analyses with four variables less than 75%. Abbreviations as in Table 2.
(XLS)

Text S1 Systematic palaeontology for *Zapsalis abradens*.
(DOC)

## References

[1] Sloan RE, Rigby JK, Van Valen L, Gabriel DL (1986) Gradual dinosaur extinction and simultaneous ungulate radiation in the Hell Creek Formation. Science 232: 629–633. 17781415 10.1126/science.232.4750.629

[2] Sheehan PM, Fastovsky DE, Hoffmann RG, Berghaus CB, Gabriel DL (1991) Sudden extinction of the dinosaurs: latest Cretaceous, Upper Great Plains, U.S.A. Science. 254: 835–839. 10.1126/science.11536489 11536489

[3] Fastovsky DE, Huang Y, Hsu J, Martin-McNaughton J, Sheehan PM, et al (2004) Shape of Mesozoic dinosaur richness. Geology 32: 877–880.

[4] Wang SC, Dodson P (2006) Estimating the diversity of dinosaurs. PNAS 103: 13601–13606. 16954187 10.1073/pnas.0606028103 PMC1564218

[5] Barrett PM, McGowan AJ, Page V (2009) Dinosaur diversity and the rock record. Proceedings of the Royal Society B 276: 2667–2674. 19403535 10.1098/rspb.2009.0352 PMC2686664

[6] Vavrek MJ, Larsson HCE (2010) Low beta diversity of Maastrichtian dinosaurs of North America. PNAS 107: 8265–8268. 20404176 10.1073/pnas.0913645107 PMC2889528

[7] Lloyd GT (2011) A refined modelling approach to assess the influence of sampling on palaeobiodiversity curves: new support for declining Cretaceous dinosaur richness. Biology Letters. doi: 10.1098/rsbl.2011.0210. 10.1098/rsbl.2011.0210 PMC3259943 21508029

[8] Russell DA, Manabe M (2002) Synopsis of the Hell Creek (uppermost Cretaceous) dinosaur assemblage. Geological Society of America Special Papers 361: 169–176.

[9] Horner JR, Goodwin MB, Myhrvold N (2011) Dinosaur census reveals abundant *Tyrannosaurus* and rare ontogenetic stages in the Upper Cretaceous Hell Creek Formation (Maastrichtian), Montana, USA. PLoS ONE 6(2): e16574 doi:10.1371/journal.pone.0016574 21347420 10.1371/journal.pone.0016574 PMC3036655

[10] Hutchison JH, Archibald JD (1986) Diversity of turtles across the Cretaceous/Tertiary boundary in northeastern Montana. Palaeogeography, Palaeoclimatology, Palaeoecology 55: 1–22.

[11] Lyson TR, Joyce WG (2010) A new baenid turtle from the Upper Cretaceous (Maastrichtian) Hell Creek Formation of North Dakota and a preliminary taxonomic review of Cretaceous Baenidae. Journal of Vertebrate Paleontology 30: 394–402.

[12] Estes R (1964) Fossil vertebrates from the Lance Formation. University of California publications in Geological Sciences 49: 1–187.

[13] Pearson DA, Schaefer T, Johnson KR, Nichols DJ, Hunter JP (2002) Vertebrate biostratigraphy of the Hell Creek Formation in southwestern North Dakota and northwestern South Dakota. Geological Society of America Special Papers 361: 145–167.

[14] Currie PJ, Rigby JK, Sloan RE (1990) Theropod teeth from the Judith River Formation of Southern Alberta, Canada. In: Carpenter K, Currie PJ, editors. Dinosaur Systematics: Approaches and Perspectives. Cambridge: Cambridge University Press. pp. 107–125.

[15] Baszio S (1997) Systematic palaeontology of isolated dinosaur tooth from the latest Cretaceous of South Alberta, Canada. Courier Forschungsinstut Senckenberg 196: 33–77.

[16] Fiorillo AR, Gangloff RA (2000) Theropod teeth from the Prince Creek Formation (Cretaceous) of northern Alaska, with speculations on arctic dinosaur paleoecology. Journal of Vertebrate Paleontology 20: 675–682.

[17] Sankey JT (2001) Late Campanian southern dinosaurs, Aguja Formation, Big Bend, Texas. Journal of Paleontology 75: 208–215.

[18] Weishampel DB, Barrett PM, Coria RA, Le Loeuff J, Xu X, et al (2004) Dinosaur Distribution. In: Weishampel DB, Dodson P, Osmólska H, editors. The Dinosauria, 2^nd^ Ed. Berkeley: University of California Press. pp. 517–606.

[19] Fanti F, Miyashita T (2009) A high latitude vertebrate fossil assemblage from the Late Cretaceous of west-central Alberta, Canada: evidence for dinosaur nesting and vertebrate latitudinal gradient. Palaeogeography, Palaeoclimatology, Palaeoecology 275: 37–53.

[20] Antunes MT, Sigogneau-Russell D (1991) Nouvelles données sur les Dinosaures du Crétacé supérieur du Portugal. Comptes Rendus de l’Académie des Sciences, Paris, 313 (Serie 11): 113–119.

[21] Antunes MT, Mateus O (2003) Dinosaurs of Portugal. Comptes Rendus Palevol 2: 77–95.

[22] Benson RBJ (2009) An assessment of variability in theropod dinosaur remains from the Bathonian (Middle Jurassic) of Stonesfield and New Park Quarry and taxonomic implications for *Megalosaurus bucklandii* and *Iliosuchus incognitos* . Palaeontology 52: 857–858.

[23] Buffetaut E, Ingavat R (1986) Unusual theropod dinosaur teeth from the Upper Jurassic of Phu Wiang, northeastern Thailand. Revue de Paléobiologie 5: 217–220.

[24] Candeiro CRA, Abranches CT, Abrantes EA, Avilla LS, Martins VC, et al (2004) Dinosaurs remains from western São Paulo state, Brazil (Bauru Basin, Adamantina Formation, Upper Cretaceous). Journal of South American Earth Sciences 18 (2004): 1–10.

[25] Christiansen P, Bonde N (2003) The first dinosaur from Denmark. Neues Jahrbuch Geol. Palaont. Abh. 227: 287–299.

[26] De Valais S, Apesteguía S (2001) Dientes asignables a Giganotosaurus (Carcharadontosauria, Theropoda) provenientes de “La Buitrera” Fm. Candeleros, provincia de Río Negro. Resúmenes XVII Jornadas Argentinas de Paleontología de Vertebrados, (Esquel). Ameghiniana. 38: 6–7R.

[27] Fanti F, Therrien F (2007) Theropod tooth assemblages from the Late Cretaceous Maevarano Formation and the possible presence of dromaeosaurids in Madagascar. Acta Palaeontologica Polonica 52: 155–166.

[28] Han FL, Clark JM, Xu X, Sullivan C, Choiniere J, et al (2011) Theropod teeth from the Middle-Upper Jurassic Shishugou Formation of northwest Xinjiang, China. Journal of Vertebrate Paleontology 31: 111–126.

[29] Park EJ (2000) Unpublished ScD thesis. Kyungpook National University, Daegu, South Korea.

[30] Poblete F, Calvo J (2003) Upper Turonian dromaeosaurid teeth from Futalognko quarry, Barreales lake, Neuquen, Patagonia, Argentina. XIX Jornldu AreenlinlS de rlleonlologrl de Verftbndos. Buenos Aires, p. 24.

[31] Prieto-Márquez A, Gaete R, Galobart A, Ardèvol L (2000) A *Richardoestesia*-like theropod tooth from the Late Cretaceous foredeep, south-central Pyrenees, Spain. Eclogae Geologicae Helvetiae 93: 497–501.

[32] Rauhut OWM (2002) Dinosaur teeth from the Barremian of Uña, Province of Cuenca, Spain. Cretaceous Research 23: 255–263.

[33] Rauhut OWM, Werner C (1995) First record of the family Dromaeosauridae (Dinosauria: Theropoda) in the Cretaceous of Gondwana (Wadi Milk Formation, northern Sudan). Paläontologische Zeitschrift 69: 475–489.

[34] Ruiz-Omeñaca JI, Canudo JI, Cuenca-Bescos G (1996) Dientes de dinosaurios (Ornitischia: Saurischia) del Barremiense superior (Cretácio inferior) de Vallipón (Castellote, Teruel). Mas de las Matas 15: 59–103.

[35] Sanz JL, Buscalioni AD, Casanovas ML, Santafe JV (1987) Dinosaurios del Cretacio Inferior de Galve (Teruel, España). Estudios Geologicos, Volumen Extraordinario Galve-Tremp, 45–64.

[36] Sweetman SC (2004) The first record of velociraptorine dinosaurs (Saurischia, Theropoda) from the Wealden (Early Cretaceous, Barremian) of southern England. Cretaceous Research 25: 353–364.

[37] Torices A, Ruiz-Omeñaca JI, Canudo I, Lopez-Martínez N (2004) Nuevos datos sobre los dinosaurios teropódos (Saurischia: Theropoda) del Cretácico superior de los Pirineos Sur-Centrales (Huesca y Lleida). Geo-Temas 6(5): 71–74.

[38] Vilas-Bôas I, Carvalho IS, Medeiros MA, Pontes H (1999) Dentes de Charcarodontosaurus (Dinosauria, Tyranosauridae) do Cenomaniano, Bacia de São Luis (Norte do Brasil). Anais da Academia Brasileira de Ciências 71: 846–847.

[39] Weigert A (1995) Isolierte Zähne von cf. Archaeopteryx sp. aus dem oberen Jura der Kohlengrube Guimarota (Portugal). Neues Jahrbuch für Geologie und Paläontologie, Monatshefte 1995: 562–576.

[40] Zinke J (1998) Small theropod teeth from the Upper Jurassic coal mine of Guimarota (Portugal). Paläontologische Zeitschrift 72: 179–189.

[41] Lehman TM (2001) Late Cretaceous dinosaur provinciality. In: Tanke DH, Carpenter K, editors. Mesozoic Vertebrate Life. Bloomington: Indiana University Press. pp. 310–328.

[42] Currie PJ (2003) Cranial anatomy of tyrannosaurid dinosaurs from the Late Cretaceous of Alberta, Canada. Acta Palaeontologica Polonica 48: 191–226.

[43] Ryan MJ, Evans DC (2005) Ornithischian dinosaurs. In: Currie PJ, Koppelhus EB, editors. Dinosaur Provincial Park: A Spectacular Ancient Ecosystem Revealed. Bloomington: Indiana University Press. pp. 312–348.

[44] Sullivan RM, Lucas SG (2006) The Kirtlandian Land-Vertebrate “Age”–Faunal composition, temporal position and biostratigraphic correlation in the nonmarine upper Cretaceous of western North America. New Mexico Museum of Natural History and Science Bulletin 35: 7–29.

[45] Smith JB, Vann DR, Dodson P (2005) Dental morphology and variation in theropod dinosaurs: implications for the taxonomic identification of isolated teeth. The Anatomical Record 285A: 699–736. 10.1002/ar.a.20206 15986487

[46] Larson DW (2008) Diversity and variation of theropod dinosaur teeth from the uppermost Santonian Milk River Formation (Upper Cretaceous), Alberta: a quantitative method supporting identification of the oldest dinosaur tooth assemblage in Canada. Canadian Journal of Earth Sciences 45: 1455–1468.

[47] Hammer Ø, Harper D (2006) Paleontological Data Analysis. Oxford: Blackwell Publishing. 368 p.

[48] Archibald JD (1997) Extinction. In: Currie PJ, Padian K, editors. Encyclopedia of Dinosaurs. San Diego: Academic Press. pp. 221–236.

[49] Lillegraven JA, McKenna MC (1986) Fossil mammals from the “Mesaverde” Formation (Late Cretaceous, Judithian) of the Bighorn and Wind River basins, Wyoming, with definitions of Late Cretaceous North American Land-Mammal “Ages.”. American Museum Novitates 2840: 1–68.

[50] Sankey JT, Brinkman DB, Guenther M, Currie PJ (2002) Small theropod and bird teeth from the Late Cretaceous (Upper Campanian) Judith River Group, Alberta. Journal of Paleontology 76: 751–763.

[51] Sankey JT, Standhardt BR, Schiebout JA (2005) Theropod teeth from the Upper Cretaceous (Campanian–Maastrichtian), Big Bend National Park, Texas. In: Carpenter K, editor. The Carnivorous Dinosaurs. Bloomington: Indiana University Press. pp. 127–152.

[52] Currie PJ, Varricchio DJ (2004) A new dromaeosaurid from the Horseshoe Canyon Formation (Upper Cretaceous) of Alberta, Canada. In: Currie PJ, Koppelhus EB, Shugar MA, Wright JL, editors. Feathered Dragons: Studies on the Transition from Dinosaurs to Birds. Bloomington: Indiana University Press. pp. 112–132.

[53] Sankey JT (2008) Diversity of latest Cretaceous (Late Maastrichtian) small theropods and birds: teeth from the Lance and Hell Creek formations, USA. In: Sankey JT, Baszio S, editors. Vertebrate Microfossil Assemblages: Their Role in Paleoecology and Paleobiogeography. Bloomington: Indiana University Press. pp. 117–134.

[54] Farlow JO, Brinkman DL, Abler WL, Currie PJ (1991) Size, shape, and serration density of theropod dinosaur lateral teeth. Modern Geology 16: 161–198.

[55] Longrich NR (2008) Small theropod teeth from the Lance Formation of Wyoming, USA. In: Sankey JT, Baszio S, editors. Vertebrate Microfossil Assemblages: Their Role in Paleoecology and Paleobiogeography. Bloomington: Indiana University Press. pp. 135–158.

[56] Buckley LG, Larson DW, Reichel M, Samman T (2010) Tooth and consequences: quantifying variation of teeth within a single population of *Albertosaurus sarcophagus* (Theropoda: Tyrannosauridae) from the Horseshoe Canyon Formation (Upper Cretaceous: lower Maastrichtian) and implications for identifying isolated tyrannosaurid teeth in Maastrichtian-aged deposits. Canadian Journal of Earth Sciences 49: 1227–1251.

[57] JMP. JMP Version 5 (2002) SAS Institute Inc., Cary, North Carolina, USA.

[58] Longrich NR, Currie PJ (2009) A microraptorine (Dinosauria–Dromaeosauridae) from the Late Cretaceous of North America. PNAS 106: 5002–5007. 19289829 10.1073/pnas.0811664106 PMC2664043

[59] Norell MA, Clark JM, Turner AH, Makovicky PJ, Barsbold R, et al (2006) A new dromaeosaurid theropod from Ukhaa Tolgod (Ömnögov, Mongolia). American Museum Novitates 3545: 1–50.

[60] Godefroit P, Currie PJ, Hong L, Yong SC, Zhi-Ming D (2008) A new species of *Velociraptor* (Dinosauria: Dromaeosauridae) from the Upper Cretaceous of northern China. Journal of Vertebrate Paleontology 28: 432–438.

[61] R Development Core Team (2011) R: A language and environment for statistical computing. R Foundation for Statistical Computing, Vienna, Austria. ISBN 3-900051-07-0, URL http://www.R-project.org/.

[62] Venables WN, Ripley BD (2002) Modern Applied Statistics with S. Fourth Edition. Springer. 497 p.

[63] Samman T, Powell GL, Currie PJ, Hills LV (2005) Morphometry of the teeth of western North American tyrannosaurids and its applicability to quantitative classification. Acta Palaeontologica Polonica 50: 757–776.

[64] Burnham DA (2004) New information on *Bambiraptor feinbergi* (Theropoda: Dromaeosauridae) from the Late Cretaceous of Montana. In: Currie PJ, Koppelhus EB, Shugar MA, Wright JL, editors. Feathered Dragons: Studies on the Transition from Dinosaurs to Birds. Bloomington: Indiana University Press. pp. 67–111.

[65] Cope ED (1876) On some extinct reptiles and Batrachia from the Judith River and Fox Hills beds of Montana. Proceedings of the Academy of Natural Sciences of Philadelphia 1876: 340–359.

[66] Leidy J (1856) Notices of remains of extinct Reptiles and Fishes, discovered by Dr. F. V. Hayden in the Bad Lands of the Judith River, Nebraska Territory. Proceedings of the Academy of Natural Sciences of Philadelphia 8: 72–73.

[67] Brinkman DB, Russell AP, Eberth DA, Peng JH (2001) Vertebrate palaeocommunities of the lower Judith River Group (Campanian) of southeastern Alberta, Canada, as interpreted from vertebrate microfossil assemblages. Palaeogeography, Palaeoclimatology, Palaeoecology 213: 295–313.

[68] Currie PJ (1987) Bird-like characteristics of the jaws and teeth of troodontid theropods (Dinosauria: Saurischia). Journal of Vertebrate Paleontology 7: 72–81.

[69] Carpenter K (1982) Baby dinosaurs from the Late Cretaceous Lance and Hell Creek formations and a description of a new species of theropod. University of Wyoming Contributions to Geology 20: 123–134.

[70] Eaton JG (2006) Santonian (Late Cretaceous) mammals from the John Henry Member of the Straight Cliffs Formation, Grande Staircase-Escalante National Monument, Utah. Journal of Vertebrate Paleontology 26: 446–460.

[71] Campione NE, Evans DC (2011) Cranial growth and variation in edmontosaurs (Dinosauria: Hadrosauridae): Implications for latest Cretaceous megaherbivore diversity in North America. PLoS ONE 6(9): e25186 doi:10.1371/journal.pone.0025186 21969872 10.1371/journal.pone.0025186 PMC3182183

[72] Larson DW, Brinkman DB, Bell R (2010) Faunal assemblages from the upper Horseshoe Canyon Formation, an early Maastrichtian cool-climate assemblage from Alberta, with special reference to the *Albertosaurus sarcophagus* bonebed. Canadian Journal of Earth Sciences 49: 1159–1181.

[73] Smith JB (2007) Dental morphology and variation in *Majungasaurus crenatissimus* (Theropoda: Abelisauridae) from the Late Cretaceous of Madagascar. Journal of Vertebrate Paleontology 27: 103–126.

