# Supplementary material for: Multivariate Analyses of Small Theropod Dinosaur Teeth and Implications for Paleoecological Turnover through Time
Source: PLoS One. 2013 Jan 23;8(1):e54329. doi: 10.1371/journal.pone.0054329 (PMC3553132; doi:10.1371/journal.pone.0054329)
Supplement: Text S1 — Systematic palaeontology for Zapsalis abradens. (DOC) [file pone.0054329.s003.doc]

SYSTEMATIC PALAEONTOLOGY

SAURISCHIA Seeley 1888

MANIRAPTORA Gauthier 1986

DROMAEOSAURIDAE Matthew and Brown 1922

EUDROMAEOSAURIA Longrich and Currie 2009

DROMAEOSAURINAE Matthew and Brown 1922

*ZAPSALIS* Cope 1876

**Type species**: *Zapsalis abradens* Cope 1876.

**Referred species**: Milk River cf. *Zapsalis* sp.

**Occurrence**: Late Cretaceous (Late Santonian–Late Maastrichtian), Alberta, Canada, and Montana, USA.

**Revised diagnosis**: (revised from Cope 1876; Baszio 1997; Sankey et al. 2002) Dromaeosaurid with large rounded denticles (usually only on the posterior carina) on the teeth, with crown morphology characterized by a long FABL relative to CH and a straight posterior margin in lateral view relative to other dromaeosaurs and one flattened (presumably lingual) side, forming a semicircle in cross-section. There are also prominent longitudinal *Paronychodon*-like ridges on both medial and lateral sides.

**Remarks**: Previously regarded as a junior subjective synonym of *Paronychodon lacustris* (Estes 1964; Currie et al. 1990), Cope (1876) originally differentiated *Zapsalis* from *Paronychodon* based on the presence of large posterior denticles. As Currie et al. (1990) restricted usage of *Paronychodon lacustris* to non-denticulate teeth, *Zapsalis* is a valid name for the denticulate morphology. The teeth with *Paronychodon*-like morphology and large rounded denticles were subsequently referred to ?*Dromaeosaurus* morphotype A (Sankey et al. 2002; Sankey 2008) or Dromaeosaurinae morphotype A (Larson 2008). It appears increasingly likely that these teeth represent a distinct species (Larson 2008; Sankey 2008) rather than a growth abnormality (Currie et al. 1990). However, if distinct, the historically valid name for the material is *Zapsalis*. This is especially pertinent given that the unique crown morphology and convergence of denticle size and shape across many lineages make referral to *Dromaeosaurus* (or even Saurischia as a whole) increasingly equivocal. Nevertheless, we feel that this morphology does belong to a species distinct from any other whose tooth morphology has been described, including *Paronychodon lacustris* and *Dromaeosaurus albertensis*.

Though the type species is the only named species of this genus, the Milk River cf. *Zapsalis* sp. quantitative morphotype is here referred to the genus based on possession of all characters here listed for the genus, but differs from the type species quite distinctly in all absolute quantitative measurements (ie. is smaller in size). This genus has also been documented from the Lance and Hell Creek formations of Montana (Baszio 1997; Sankey 2008), but so infrequently that no measurements were available for comparison.

*ZAPSALIS ABRADENS* Cope 1876

**Synonymies**: ?*Dromaeosaurus* morphotype A Sankey, Brinkman, Guenther, and Currie 2002

**Holotype**: AMNH FR 3953

**Referred specimens:** TMP 1979.015.0003; TMP 1980.016.0833; TMP 1982.019.0007; TMP 1982.019.0458; TMP 1982.019.0458; TMP 1984.091.0040 (two teeth); TMP 1984.163.0080; TMP 1985.006.0002; TMP 1985.006.0131; TMP 1985.006.0133; TMP 1985.058.0065; TMP 1986.036.0425; TMP 1986.077.0112; TMP 1987.036.0005; TMP 1987.050.0008; TMP 1989.036.0312; TMP 1989.050.0202; TMP 1990.050.0208; TMP 1990.053.0021; TMP 1991.050.0060; TMP 1994.012.0268; TMP 1997.133.0002.

**Occurrence:** (Holotype) Late Campanian Judith River Formation, Montana, USA; (referred specimens) Late Campanian Dinosaur Park Formation, Alberta, Canada.

**Revised diagnosis:** A species of *Zapsalis* characterized by possessing rather large teeth for the genus, measuring in currently know specimens 5–7.9 mm in FABL, 2.1–3.4 mm in BW, 9.3–15.5 mm in CH, and with 3–3.75 PDM.

**Revised description:** (revised from Cope 1876 and Sankey et al. 2002) These teeth have large, rounded, *Dromaeosaurus*-like denticles on the posterior carinae. Denticles are variably present on the anterior carinae, and, when present, are similar in size to those on the posterior carinae. In contrast to most theropod teeth, the posterior margin, in lateral view, is straight, whereas the anterior margin is curved, giving the crowns a distinct appearance in lateral view. Crowns have one convex surface and one flattened surface (probably lingual). Both labial and lingual surfaces have prominent longitudinal ridges. A distinct wear pattern is present on most teeth, with both the tip and base of the flattened surface often bearing extensive wear.

**Remarks:** Because of the stratigraphic position and near-identical nature of measurements, the Dinosaur Park Formation specimens are here referred to the same species as the holotype Judith River Formation specimen. Based on the constrained morphology and wear patterns of known specimens, these teeth are unlikely to represent a complete dentition. However, until such a time as there is more evidence other than denticle shape and size for classifying the species or relating it to other tooth morphologies, we will retain this species as distinct for ease of communication.
